# eHealth in Geriatric Rehabilitation: An International Survey of the Experiences and Needs of Healthcare Professionals

**DOI:** 10.3390/jcm12134504

**Published:** 2023-07-05

**Authors:** Jules J. M. Kraaijkamp, Anke Persoon, Sorina Aurelian, Stefan Bachmann, Ian D. Cameron, Mohamed-Amine Choukou, Frances Dockery, Kseniia Eruslanova, Adam L. Gordon, Stefan Grund, Hyub Kim, Andrea B. Maier, Laura M. Pérez Bazan, José E. Pompeu, Eva Topinkova, Mark A. Vassallo, Niels H. Chavannes, Wilco P. Achterberg, Eléonore F. Van Dam van Isselt

**Affiliations:** 1 University Network for the Care Sector Zuid-Holland, Leiden University Medical Center, 2333 ZA Leiden, The Netherlands; 2 Department of Public Health and Primary Care, Leiden University Medical Center, 2333 ZA Leiden, The Netherlands; 3 ZZG Zorggroep, 6561 LE Nijmegen, The Netherlands; 4 Department of Primary and Community Care, Radboud University Medical Center Nijmegen, 6525 GA Nijmegen, The Netherlands; 5 Department of Geriatric, University of Medicine and Pharmacy “Carol Davila”-Chronic Disease Hospital “Sf. Luca”, 041915 Bucharest, Romania; 6 Department of Internistic and Musculoskeletal Rehabilitation, Kliniken Valens, 7317 Valens, Switzerland; 7 Department of Geriatrics, Faculty of Medicine, Inselspital, University of Bern, 3010 Bern, Switzerland; 8 John Walsh Centre for Rehabilitation Research, Northern Sydney Local Health District and Faculty of Medicine and Health, University of Sydney, St Leonards, NSW 2065, Australia; 9 Department of Occupational Therapy, College of Rehabilitation Sciences, Rady Faculty of Health Sciences, University of Manitoba, Winnipeg, MB R3E 0T6, Canada; 10 Centre on Aging, University of Manitoba, Winnipeg, MB R3T 2N2, Canada; 11 Beaumont Hospital & Royal College of Surgeons in Ireland, D09 V2NO Dublin, Ireland; 12 Russian Clinical and Research Center of Gerontology, 129226 Moscow, Russia; 13 Academic Unit of Injury, Recovery and Inflammation Sciences (IRIS), School of Medicine, University of Nottingham, Nottingham NG7 2UH, UK; 14 Center for Geriatric Medicine, Agaplesion Bethanien Hospital Heidelberg, Geriatric Center at the Heidelberg University, 69117 Heidelberg, Germany; 15 Department of Occupational Therapy, Far East University, 76-32, Daehak-gil, Gamgok-myeon, Eumseong 27601, Republic of Korea; 16 Healthy Longevity Program, Yong Loo Lin School of Medicine, National University of Singapore, Singapore 117597, Singapore; 17 Centre for Healthy Longevity, @AgeSingapore, National University Health System, Singapore 117456, Singapore; 18 Department of Human Movement Sciences, @AgeAmsterdam, Faculty of Behavioural and Movement Sciences, Vrije Universiteit Amsterdam, Amsterdam Movement Sciences, 1081 BT Amsterdam, The Netherlands; 19 Research on Aging, Frailty and Care Transitions in Barcelona (REFiT-BCN), Parc Sanitari Pere Virgili and Vall d’Hebron Institute (VHIR), 08023 Barcelona, Spain; 20 Department of Physiotherapy, Speech Therapy and Occupational Therapy, School of Medicine, University of São Paulo, São Paulo 05360-160, Brazil; 21 Department of Geriatric Medicine, First Faculty of Medicine and General Faculty Hospital, 120 00 Prague, Czech Republic; 22 Faculty of Health and Social Sciences, South Bohemian University, 370 11 České Budějovice, Czech Republic; 23 Geriatric Medicine Society of Malta, Karin Grech Hospital, PTA 1312 Pieta, Malta

**Keywords:** geriatric rehabilitation, eHealth, implementation, barriers and facilitators, information needs

## Abstract

While eHealth can help improve outcomes for older patients receiving geriatric rehabilitation, the implementation and integration of eHealth is often complex and time-consuming. To use eHealth effectively in geriatric rehabilitation, it is essential to understand the experiences and needs of healthcare professionals. In this international multicentre cross-sectional study, we used a web-based survey to explore the use, benefits, feasibility and usability of eHealth in geriatric rehabilitation settings, together with the needs of working healthcare professionals. Descriptive statistics were used to summarize quantitative findings. The survey was completed by 513 healthcare professionals from 16 countries. Over half had experience with eHealth, although very few (52 of 263 = 20%) integrated eHealth into daily practice. Important barriers to the use or implementation of eHealth included insufficient resources, lack of an organization-wide implementation strategy and lack of knowledge. Professionals felt that eHealth is more complex for patients than for themselves, and also expressed a need for reliable information concerning available eHealth interventions and their applications. While eHealth has clear benefits, important barriers hinder successful implementation and integration into healthcare. Tailored implementation strategies and reliable information on effective eHealth applications are needed to overcome these barriers.

## 1. Introduction

With an aging global population and an ever-expanding number of older adults with one or more long-term conditions, the demands placed on geriatric rehabilitation are increasing rapidly [1]. Geriatric rehabilitation has been defined as “a multidimensional approach of diagnostic and therapeutic interventions, the purpose of which is to optimize functional capacity, promote physical activity and preserve functional reserve and social participation in older people with disabling impairments” [2]. Due to a rapidly expanding older population and an increasing lack of staff, new strategies are required to maintain and advance the implementation and delivery of geriatric rehabilitation. Promising solutions such as eHealth may be one way to help overcome these challenges.

One definition of eHealth is “the use of digital information and communication to support and/or improve health and healthcare” [3]. eHealth interventions vary widely, from simple approaches such as video communication, to complex treatment applications involving robotics. A growing body of evidence suggests that eHealth can contribute to improved outcomes for older patients receiving geriatric rehabilitation [4,5,6,7]. The COVID-19 pandemic highlighted the need for substantial changes in the delivery of rehabilitation, with reduced capacity, reduced time spent per patient and reduced access to rehabilitation facilities [8,9]. This emphasizes the importance of eHealth interventions that enable remote monitoring and treatment of patients, enhancing the accessibility and the continuity of rehabilitation. Although the COVID-19 pandemic has accelerated the use of eHealth, the adoption of eHealth is still lagging behind and a number of obstacles hinder the successful development, implementation and integration of eHealth in geriatric rehabilitation [10].

Successful implementation of eHealth involves considerable time and effort and is often complex [11,12,13]. To facilitate integration into clinical practice, implementation of eHealth may also require changes to a healthcare professional’s workflow [14,15]. Another challenge facing healthcare professionals is the ever-increasing number of eHealth interventions and staying up to date in which eHealth interventions are effective, feasible, usable and suit their specific context [16,17].

As healthcare professionals are central to the successful application of eHealth, the key to promoting implementation and integration of eHealth in geriatric rehabilitation is a better understanding of the experiences and needs of healthcare professionals. The goal of this study was to provide an overview of the use, benefits, feasibility, usability and needs of healthcare professionals regarding eHealth in geriatric rehabilitation across different countries. This study is part of the EAGER (EheAlth in GEriatric Rehabilitation) research line. The first study consisted of a systematic review of the effectiveness, feasibility and usability of eHealth in geriatric rehabilitation [4].

## 2. Materials and Methods

### 2.1. Design

An online international multicentre cross-sectional survey study was conducted between December 2021 and April 2022. Results were reported based on the Checklist for Reporting Results of Internet E-Surveys (CHERRIES), a 30-item checklist for web surveys [18].

### 2.2. Study Population and Setting

We included healthcare professionals who were (1) working in a geriatric rehabilitation setting, (2) aged 18 years old and over, (3) understood English and (4) had at least three months experience with the patient population. Healthcare professionals not available during the study period were excluded. Taking into account international variation between different healthcare systems and provision of geriatric rehabilitation [19,20], we included a range of geriatric rehabilitation settings such as post-acute rehabilitation facilities, acute hospitals, ambulatory settings, geriatric day hospitals, nursing homes, skilled nurse facilities and rehabilitation hotels.

### 2.3. Recruitment and Consent

Eligible healthcare professionals were recruited in geriatric rehabilitation facilities across 16 countries: Australia, Brazil, Canada, Czech Republic, Germany, Ireland, Malta, The Netherlands, New Zealand, Romania, Russia, Singapore, South Korea, Spain, Switzerland and the United Kingdom. Per country, one primary contact person was designated to distribute the survey to the geriatric rehabilitation facilities within that country. All primary contacts were experts in the field of geriatric rehabilitation and/or eHealth and were native speakers. Almost all persons acting as primary contacts were members of the European Geriatric Medical Society’s ‘Special Interest Group for Geriatric Rehabilitation’ and were recruited through this network. Distribution of the survey varied per country, based on the personal preferences and experiences of the primary contact. Distribution variously consisted of email lists, posts to specific professional societies (such as the British Geriatrics Society) and posts on social networks (Twitter and LinkedIn). The survey invitation included a link to the online survey and study information including purpose, expected duration (10 min), voluntariness of participation, confidentiality of responses and contact details of the principal investigator. To increase response rates, in each participating country a reminder was sent two weeks after the initial invitation.

### 2.4. Data Collection

A digital survey was designed based on the experiences of experts in eHealth in geriatric rehabilitation and the results of our previous systematic review on eHealth in geriatric rehabilitation [4]. We designed the first draft in Dutch and piloted it in a national study within the Netherlands. The first draft consisted of a total of 24 questions, four of which were open-ended to obtain detailed information. To improve accuracy and reliability of data analysis, the results of these open-ended questions were indexed and converted into multiple-choice questions. The second draft was then translated into English and sent to our primary contacts in each country for feedback. Based on their suggestions for improvement, the survey was revised with the goal of ensuring an adequate balance between the existing and the revised or new questions. The main changes entailed the phrasing of the questions and questions related to specific eHealth interventions. In the final version of the survey, questions could only be answered by participants who had experience with that type of eHealth intervention. The final survey was then translated into six languages (Czech, German, Portuguese, Romanian, Russian and Spanish) by the primary contact person in the corresponding country. The online survey was hosted by Castor Electronic Data Capture (Castor EDC; Castor, Amsterdam, The Netherlands) [21], a secure, cloud-based electronic data capture platform. The survey had a maximum of 10 questions per page, all of which were mandatory. If a respondent failed to complete a particular question, they were asked to complete it before they moved on to the next section. Respondents could review and edit answers at any time during completion of the survey. No personal information was collected and no participant IP addresses were stored or downloaded.

### 2.5. Measures

The survey was divided into six sections: participant characteristics, use of eHealth, benefits, usability, feasibility and the needs of professionals regarding eHealth in geriatric rehabilitation. Questions in the sections regarding benefits, usability and feasibility only became visible to respondents who indicated that they had used eHealth during their treatments. Respondents who indicated that they had used specific types of eHealth interventions were asked about their experience regarding benefits and usability for each type of eHealth. The final survey consisted of 33 questions. All questions were structured and were multiple-choice or scale questions. The scale questions were formulated as follows: For each type of eHealth intervention, respondents were asked to rate the ease of use for the professional and the patient based on a 5-point Likert scale (1 = very complex to 5 = very easy). Respondents were asked how satisfied they were with the implementation of eHealth in their institution based on a 100-point scale (0 = very dissatisfied to 100 = very satisfied). Lastly, respondents were asked to rate their institution’s vision regarding the use of eHealth on a 100-point scale (0 = inadequate to 100 = good).

### 2.6. Statistical Analyses

Descriptive statistics and frequency distributions were used to describe the single-choice and multiple-choice questions. A Pearson product–moment correlation was run to determine the relationship between satisfaction with the implementation of eHealth and the vision of the use of eHealth in the corresponding institution. A one sample t-test was run to determine the difference between mean scores for ease of use of all types of eHealth interventions. A heatmap was created for results related to the benefits of eHealth, with results classified and color-coded from red (0%) to green (100%). Surveys less than 90% complete were excluded from the final data analysis. Data were analyzed with SPSS version 25.0.

### 2.7. Ethical Considerations

This study was approved by the Medical Ethics Review Committee of Leiden–Den Haag–Delft (N20.126.1) and approved by the relevant ethics committee in participating countries as per local requirements. All participants signed the informed e-consent by clicking a dedicated button available in the invitation link, with which they stated that they were aware that participation was voluntary.

## 3. Results

Overall, the survey was initialized 794 times, with 513 (65%) participants completing 90% or more of the survey questions. Participant characteristics are presented in Table 1. The majority were from Europe (439 of 513 = 86%), of whom most were from The Netherlands (248 of 513 = 48%) or the Czech Republic (52 of 513 = 10%). The median age of participants was 39 years (IQR 32–49), the median number of years of work experience within geriatric rehabilitation was 8 (IQR 4–15) and 64% (329 of 513) of the respondents were female. Participants mostly worked as physiotherapists (163 of 513 = 33%), medical practitioners/geriatricians (107 of 513 = 22%) or as nurses (82 of 513 = 17%).

### 3.1. Use of eHealth

Results for the use of eHealth are presented in Table 2. Just over half of the respondents (263 of 513 = 51%) reported using eHealth during their treatments. Of the participants with experience in eHealth during their treatments, only a small proportion (20%) used eHealth daily or almost daily. Overall, only a small percentage of the total number of participants included in this study used eHealth daily or almost daily (52 of 513 = 10%). We also found wide variation between countries in terms of experience with eHealth (ranging from 35% to 94%) and the daily use of eHealth (ranging from 2% to 56%). Of the 263 participants with experience in eHealth, a substantial number had used simple interventions such as mobile apps (153 of 263 = 58%) and video consultations with patients (140 of 263 = 53%). More complex eHealth interventions, such as robotics (42 of 263 = 16%) or virtual reality (36 of 263 = 14%) were used far less often. A little less than half of the participants who responded to questions concerning training in the use of eHealth (78 of 160 = 49%) had received some form of training.

### 3.2. Benefits

The benefits experienced per form of eHealth are described in Table 3. Most participants who had experience with specific types of eHealth indicated that virtual reality (20 of 26 = 77%), exergames (29 of 50 = 73%) and robotics (28 of 36 = 78%) improved the rehabilitation environment. These participants also felt that virtual reality (23 of 26 = 64%), exergames (28 of 50 = 70%) and robotics (28 of 36 = 78%) increased patients’ self-management. Almost all participants who had used video consultation for contact with patients (61 of 68 = 90%) indicated that it was beneficial for remote care.

Regarding specific patient benefits, most participants with experience in robotics (23 of 36 = 64%) indicated that it helped a faster recovery. Of the participants with experience in video consultations, 68% (46 of 68) indicated that it contributed to increasing the frequency of treatment. Similarly, of the participants with experience in exergames, 62% (25 of 40) stated that it increased patients’ confidence, while 62% (29 of 47) of participants with experience in health sensors perceived increased self-direction amongst patients during treatment. Participants also indicated that virtual reality (19 of 26 = 79%), exergames (36 of 40 = 90%) and robotics (25 of 36 = 69%) offered the patient a more entertaining form of therapy.

### 3.3. Feasibility

Outcomes concerning feasibility are presented in Table 4. Participants who had previously used eHealth were asked about problems they may have encountered during regular use. The most frequently reported problems were (1) insufficient available resources (89 of 136 = 65%), (2) no organization-wide method of working or implementation (69 of 136 = 51%) and (3) costs (58 of 136 = 43%). Participants reported certain risks associated with using eHealth, such as technical problems (105 of 136 = 77%), no supervision (58 of 136 = 43%) and concerns regarding the replacement of physical contact (57 of 136 = 42%). When asked to rate the implementation process of eHealth within their department, participants reported low satisfaction with the implementation of eHealth within their settings (median 40, IQR 4.0–63), while only 11% (15 of 136) indicated they were very satisfied (range 75–100).

### 3.4. Usability

**Figure 1:**
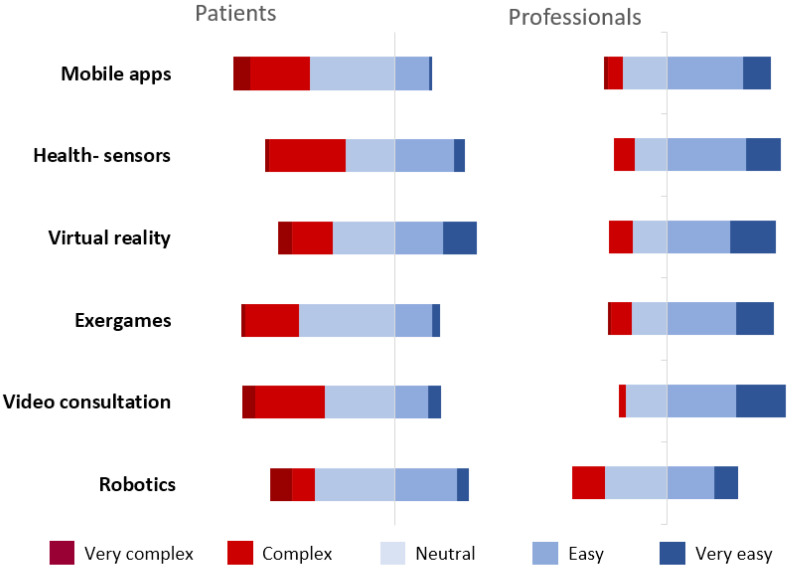
Usability: ease of use per form of eHealth, as perceived by professionals. Distribution of the ease-of-use scales per form of eHealth, ranging from very complex to very easy.

The ease of use per type of eHealth intervention is displayed in Figure 1. According to healthcare professionals, patients found virtual reality (median 3, IQR 2–4) and robotics (median 3, IQR 3–4) the most easy-to-use eHealth interventions. For professionals themselves, video consultations (median 4, IQR 3–5) and virtual reality (median 4, IQR 3–5) were the most easy-to-use forms of eHealth. Mobile apps were felt to be the most complex type to use by patients (median 3, IQR 2–4) and as the second most complex by professionals (median 3, IQR 2–3), with professionals rating robotics as the most complex form (median 3, IQR 3–4). With the exception of robotics (*p* = 0.208), professionals found all types of eHealth interventions significantly easier to use compared with patients (*p* ≤ 0.01).

### 3.5. Needs

**Figure 2:**
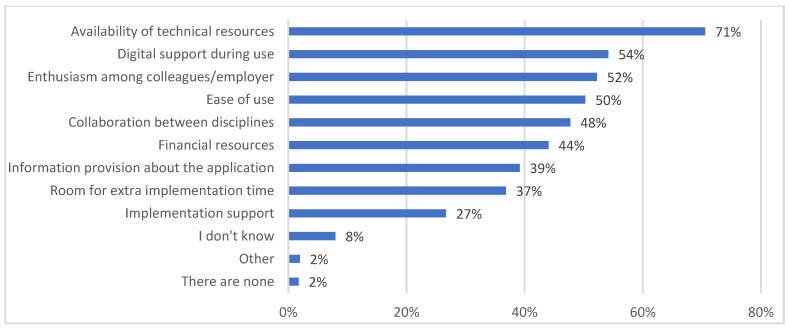
Enabling factors that influence the use or implementation of eHealth (*n* = 513).

**Figure 3:**
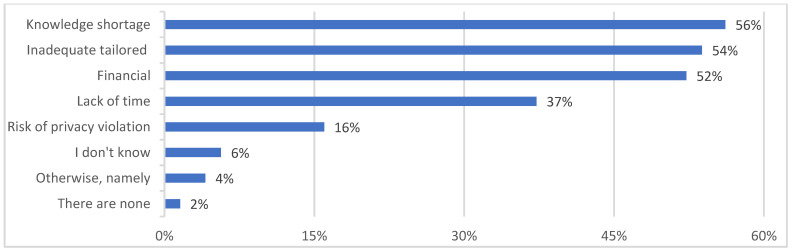
Barriers that influence the use or implementation of eHealth (*n* = 513).

Enabling factors and barriers to the use or implementation of eHealth are presented in Figure 2 and Figure 3. Results for the analysis of professional needs are described in Table 5. The majority of participants indicated that the availability of technical resources (362 of 513 = 71%), digital support during use (278 of 513 = 54%), enthusiasm among colleagues/employers (268 of 513 = 52%) and ease of use (258 of 513 = 50%) were enabling factors that influenced the use or implementation of eHealth. By contrast, lack of knowledge (288 of 513 = 56%), inadequate tailoring to the older population in geriatric rehabilitation (276 of 513 = 54%) and financial issues (268 of 513 = 52%) were considered barriers to the use or implementation of eHealth.

According to participants, the most important information was related to the types of eHealth available (381 of 513 = 74%), the application or implementation of eHealth (355 of 513 = 69%) and the benefits of eHealth (311 of 513 = 61%). Fifty-eight percent (297 of 513) of participants indicated they would like to increase their use of eHealth. The odds of participants who had experience with eHealth considering making more use of eHealth was 3.135 (95% CI 2.19, 4.47) times compared to participants who did not have any experience with eHealth. Participants rated their institution’s vision regarding the use of eHealth as inadequate (median: 25, IQR 3–50) and only 8% (22 of 265) held a positive view of their institution’s vision (range 75–100) concerning the use of eHealth. There was a strong correlation between satisfaction with the implementation of eHealth and a clear institutional vision regarding the use of eHealth (r = 0.716, *p* ≤ 0.01).

## 4. Discussion

### 4.1. Principal Findings

This international survey provided an overview of the use, benefits, feasibility, usability and needs of healthcare professionals regarding eHealth in geriatric rehabilitation. The survey included 513 professionals working in geriatric rehabilitation facilities across 16 countries. This large study is the first regarding eHealth in this setting. First, while over half of all participating healthcare professionals had experience of eHealth in clinical practice, only a tiny percentage (20%) integrated eHealth into their daily practice. Second, an institution-wide strategy for the use and implementation of eHealth (that includes topics such as the availability of technical resources, digital support and training) is an important enabling factor for the successful use and implementation of eHealth. Third, according to healthcare professionals, patients find eHealth complex to use, especially patients with cognitive impairment. Finally, there is a considerable need among professionals for more information concerning available and effective eHealth interventions, together with how they can be best applied and implemented.

### 4.2. Comparison with Prior Work

Overall, the healthcare professionals involved in this study reported a low daily use of eHealth interventions. To the best of our knowledge (based on literature available in English), this is the first study investigating the use of eHealth in geriatric rehabilitation. Previous studies that examined the use of eHealth in other healthcare settings such as home care [22], inpatient rehabilitation centers [23,24], or primary care [25,26,27] reported moderate (43%) to low (13%) use of eHealth. However, the reported use of eHealth varied greatly, dependent on the publication date, type of eHealth and countries included. Furthermore, the frequency of use was not reported. Variation between countries can potentially be explained by factors ranging from the healthcare professional’s personal characteristics such as attitudes toward digital technology, personal experience with eHealth interventions, trust in eHealth interventions or demographics [26,27,28], to regional factors such as readiness of a healthcare system, as well as policy and cultural differences [29].

Although daily use of eHealth was low, most respondents expressed a willingness to increase their use. Our results identified several barriers to increasing the structured use of eHealth in geriatric rehabilitation, including insufficient availability of resources, the lack of an organization-wide implementation strategy and a lack of knowledge. Similar barriers, including a limited knowledge of eHealth, lack of resources and the lack of integration into the daily workflow, have been reported in earlier studies [13,30,31]. Conversely, an institution-wide strategy that includes topics such as the availability of technical resources and digital support for the use of eHealth are important enabling factors that increase the structured use of eHealth. Earlier reviews that examined barriers and facilitators influencing the implementation and integration of eHealth arrived at similar conclusions [13,22,30,32,33], with regularly identified facilitators including ease of use, leadership engagement and adaptability of eHealth [13,30,31]. Although these earlier studies were not conducted in a geriatric rehabilitation setting, the commonality of results suggests that barriers and facilitators influencing implementation and integration of eHealth are likely generalizable across different healthcare settings. However, these studies also noted that barriers are dynamic and likely to change over time [30]. Finally, while the COVID-19 pandemic accelerated the use of eHealth, healthcare systems still face challenges when attempting to adopt eHealth, primarily due to difficulty in adjusting workflows and a funding system geared to delivering face-to-face care [10].

According to the professionals participating in our study, their patients find eHealth complex to use, although this varied considerably depending on the eHealth intervention and professionals might underestimate patients’ capabilities. Age-related impairments such as the cognitive, physical and visual limitations that are common in older adults can greatly influence one’s ability to effectively use eHealth interventions [34,35,36,37]. Furthermore, in a recent review, we identified studies with exclusion rates of up to 80%, with cognitive impairment as the most commonly reported reason for exclusion [4]. This is in line with our present findings, since adequate cognitive functioning as well as adequate vision, hearing or speech are all frequently reported requirements if patients are to make effective use of eHealth. Of the available eHealth interventions, patients find mobile apps the most complex type according to healthcare professionals. Although mobile apps are usually widely available and easily downloadable from an app store, few apps have been developed using a co-creation process or fewer still are sufficiently tailored to the age-related impairments of an older adult receiving geriatric rehabilitation [33,38]. Finally, while healthcare professionals viewed eHealth as complex for patients, the complexity for healthcare professionals should not be underestimated. Ease of use is the most frequently cited factor underlying successful use of eHealth by healthcare professionals, making it a key prerequisite for the implementation and integration of eHealth [13,22,30].

Our results also indicated that the majority of respondents are willing to make greater use of eHealth. However, it should be noted that acceptance of eHealth by healthcare professionals may differ in daily practice, since previous studies have found a limited acceptance of eHealth [17,23,39]. Acceptance is often based on prior experience, added value and social support for eHealth from colleagues [22,40,41]. Barriers can be overcome with continuing education for healthcare professionals, a modernized education of healthcare students that includes eHealth awareness, as well as co-creation and behavior change techniques that should be part of any implementation strategy [23,28].

In the survey, respondents indicated a need for reliable information on types of available eHealth interventions, how they might be applied and the benefits they may have. These findings support existing literature which stresses the urgent need to provide healthcare providers with information on both effective and ineffective eHealth applications, as well as those that might suit their local context [32,42]. Due to a rapidly changing landscape of eHealth applications, in which eHealth interventions are constantly added, updated or deleted, it is difficult for professionals to remain up to date, to determine which eHealth interventions are easy to use for older adults and to understand the assessed criteria. Our findings on benefits and usability indicate which types of eHealth interventions are easier to use or are suitable, for example, for improving the rehabilitation environment or increasing patients’ self-management. Nevertheless, we do not provide a comprehensive overview. Assessment frameworks of eHealth interventions, such as the CEN-ISO/TS 82304-2 can keep pace with the development of eHealth interventions and may help healthcare professionals obtain the information necessary for informed decision making [43,44].

### 4.3. Strengths and Limitations

An important strength of this study was the process of survey creation, which was comprehensive and had both valuable and executable aspects, improving the accuracy and reliability of the data analysis. Another strength of the survey was the inclusion of 513 respondents from 16 countries. This provided a good overview of the use and experiences of healthcare professionals regarding eHealth in geriatric rehabilitation. Nonetheless, some limitations of the study should be mentioned. While the study provided a broad view across a range of countries, the number of participants per country varied considerably and the majority of participants were from countries within Europe, in particular from The Netherlands and Czech Republic. This inevitably led to less reliable data for those countries with fewer respondents. Furthermore, due to the iterative development of the survey, some questions were only visible to participants outside The Netherlands, making comparisons between countries difficult. Therefore, while our paper presents the trends observed in data collected from 16 countries, our conclusions do not necessarily apply to all the countries cited in this paper. Lastly, the focus of this study was on the perspective of healthcare professionals. Future studies with a larger focus on the perspective of older adults receiving geriatric rehabilitation are needed to explore this key stakeholder’s voice.

## 5. Conclusions

Our primary conclusions are (1) eHealth is not yet sufficiently integrated in geriatric rehabilitation, (2) an institution-wide strategy that addresses context-specific barriers and facilitators is critical for the successful use and implementation of eHealth, (3) eHealth interventions that are simple, tailored and preferably developed through a co-creation process are essential, especially for older adults who suffer from cognitive impairment and (4) there is an urgent need to support healthcare providers by offering training and information on how to identify, assess and use eHealth, as well as how to evaluate implementation. Future studies on this topic should focus more on greater geographic diversity, including the views and attitudes of older adults receiving geriatric rehabilitation in various contexts, as well as take account of individual characteristics such as attitudes towards eHealth, gender, ethnicity, education and social network. These studies are preferably conducted using qualitative methods, such as in-depth interviews or focus groups. Furthermore, as assessment frameworks such as the CEN-ISO/TS 82304-2 are more widely adopted, it is advisable that these frameworks are tailored to geriatric rehabilitation via a greater emphasis on usability and specific age-related limitations.

## Figures and Tables

**Table 1:** Sociodemographic and professional characteristics of participants (*n* = 513).

	*n* (%)
**Sex**	
Female	329 (64)
Male	178 (35)
Prefer not to say	5 (1)
**Age**	
18–29	100 (20)
30–39	158 (31)
40–49	130 (25)
50–59	83 (16)
>60	42 (8)
**Profession**	
Physiotherapist	163 (33)
Medical practitioner/geriatrician	107 (22)
Nurse	82 (17)
Occupational therapist	61 (13)
Speech therapist	29 (6)
Other	74 (15)
**Working years**	
0–5	171 (33)
6 to 15	218 (43)
16 to 25	92 (18)
>25	32 (6)
**Continent**	
Europe (including the United Kingdom and Ireland)	439 (86)
Asia	50 (10)
North and South America	32 (6)
Oceania	10 (2)
**Type of rehabilitation facility**	
Post-acute rehabilitation facility	342 (67)
Acute hospital	45 (9)
Ambulatory (home based)	39 (8)
Geriatric day hospital	38 (7)
Other	49 (10)
**Experience with eHealth during treatments?**	
Yes	263 (51)
No	250 (49)

Profession, other: Nurse practitioner physician assistant, medical practitioner in training, psychologist, dietician, manager/team leader, researcher, social worker. Type of rehabilitation facility, other: Nursing home, skilled nursing facility, rehab hotel.

**Table 2:** Frequency of the use of eHealth (*n* = 263).

	*n* (%)
**Applied types of eHealth interventions**	
Mobile apps	153 (58)
Video consultation with patients	140 (53)
Health sensors	101 (38)
Exergames	101 (38)
Robotics	42 (16)
Virtual reality	36 (14)
**Frequency of use**	
Incidental	92 (35)
Weekly	65 (25)
Few times a month	54 (21)
Daily or almost daily	52 (20)
**eHealth part of a rehabilitation program**	
Yes, right now	143 (51)
Yes, in the past	36 (13)
No	91 (33)
I don’t know	8 (3)
**Training received in the use of eHealth (*n* =160)**	
No, I’ve only read the included manual	52 (33)
No	29 (18)
Yes, I received training on how to use eHealth	40 (25)
Yes, I received training on the implementation of eHealth	22 (14)
Yes, I received training on how to tailor eHealth to age-related barriers (e.g., cognitive or physical disabilities)	16 (10)
I don’t know	1 (1)

**Table 3:** Heatmap of benefits per form of eHealth.

	Mobile Apps *n* = 75	Health Sensors *n* = 47	Virtual Reality *n* = 26	Exergames *n* = 40	Video Consultation *n* = 68	Robotics *n* = 36
**Types of benefits experienced**						
Ease of use	24 (32%)	18 (38%)	6 (23%)	10 (25%)	26 (38%)	9 (25%)
Better quality treatment	36 (47%)	23 (49%)	6 (23%)	10 (25%)	30 (44%)	7 (9%)
Improvement of the rehabilitation environment	23 (30%)	17 (36%)	20 (77%)	29 (73%)	18 (26%)	28 (78%)
Increasing self-management of the patient	21 (28%)	14 (30%)	18 (69%)	28 (70%)	8 (12%)	23 (64%)
Possibility of remote care	43 (57%)	19 (50%)	6 (23%)	7 (18%)	61 (90%)	2 (18%)
Efficient deployment of staff	37 (49%)	23 (49%)	9 (35%)	17 (43%)	30 (44%)	12 (33%)
**Types of benefits for the patient**						
Faster recovery	14 (19%)	13 (28%)	13 (54%)	18 (45%)	10 (15%)	23 (64%)
Increase in treatment frequency	39 (52%)	15 (32%)	12 (50%)	18 (45%)	46 (68%)	13 (36%)
More confidence	38 (51%)	26 (55%)	12 (50%)	25 (62%)	25 (37%)	11 (31%)
More self-direction	40 (53%)	29 (62%)	10 (42%)	17 (42%)	32 (47%)	11 (31%)
More fun form of therapy	30 (40%)	15 (32%)	19 (79%)	36 (90%)	15 (22%)	25 (69%)
**Types of disadvantages encountered**						
It does not meet the needs of the patient well	28 (39%)	14 (30%)	3 (16%)	12 (31%)	26 (40%)	10 (29%)
Applications crash or do not work properly	22 (31%)	11 (24%)	4 (21%)	10 (26%)	25 (39%)	6 (18%)
Difficult to use or apply	30 (42%)	18 (39%)	3 (16%)	16 (31%)	19 (29%)	17 (50%)
None	16 (22%)	14 (30%)	10 (53%)	13 (33%)	16 (25%)	12 (35%)

**Color code:**
0%




100%

**Table 4:** Results related to feasibility of eHealth (*n* = 136).

	*n* (%)
**Problems encountered in structural use of eHealth**	
Insufficient available resources	89 (65)
No organization-wide method of working/implementation	69 (51)
Costs	58 (43)
Shortage of professional knowledge	55 (40)
Lack of time	47 (34)
Space shortage	37 (27)
Inappropriate target group	26 (19)
Lack of motivation	20 (15)
Other (adherence, accessibility, lack of effort)	10 (7)
No problems	5 (4)
**Patient‘s skills to use eHealth**	
Sufficient cognitive functioning	112 (82)
No problems with vision, hearing or speech	77 (57)
Supervision from caregiver/family	72 (53)
Motivation	67 (49)
Independence	57 (42)
Digital literacy	56 (41)
Sufficient motor functioning	24 (18)
**Risks of eHealth**	
Technical problems	105 (77)
No supervision	58 (43)
Concerns regarding replacement of physical contact	57 (42)
Distress/confusion in patients	53 (39)
Difficult to implement	52 (38)
Reduction in quality of care	39 (29)
Privacy sensitive	31 (23)
Discomfort (i.e., lower back pain, lower limb pain)	22 (16)
Other (no risks, solitude, digital literacy, poor performance exercises)	10 (3)

**Table 5:** Needs of professionals regarding the use of eHealth (*n* = 513).

	*n* (%)
**Information needs concerning eHealth**	
Which types of eHealth exist	381 (74)
Applying or implementing eHealth	355 (69)
The benefits of eHealth	311 (61)
The operation of eHealth applications	260 (51)
I don’t have any information needs	23 (4)
I don’t know	13 (3)
Other (training)	6 (1)
**How to receive information about eHealth (*n* = 265)**	
Digital course	140 (53)
Course on location	137 (52)
Webinar	129 (49)
Written (article, information letter, manual)	100 (38)
Fact sheet	76 (29)
No preference	18 (7)
**Would you like to make more use of eHealth?**	
Yes	297 (58)
Maybe	160 (31)
I don’t know	41 (8)
No	15 (3)

## Data Availability

The data presented in this study are available upon reasonable request from the corresponding author. Requests will be judged based on the originality of the research question and the feasibility of the analysis plan.
